# Treatment and prognosis of colorectal cancer with synchronous peritoneal metastases: 11-year single institute experience

**DOI:** 10.1136/egastro-2023-100016

**Published:** 2023-09-20

**Authors:** Xiusen Qin, Zifeng Yang, Yang Li, Jian Luo, Hui Wang, Huaiming Wang

**Affiliations:** 1 Department of General Surgery, The Sixth Affiliated Hospital, Sun Yat-sen University, Guangzhou, Guangdong, China; 2 Guangdong Institute of Gastroenterology, Guangdong Provincial Key Laboratory of Colorectal and Pelvic Floor Diseases, The Sixth Affiliated Hospital, Sun Yat-sen University, Guangzhou, Guangdong, China; 3 Department of General Surgery, Guangdong Provincial People's Hospital, Guangdong Academy of Medical Sciences, Guangzhou, Guangdong, China

**Keywords:** Colorectal neoplasms

## Abstract

**Background:**

Treatment of colorectal cancer (CRC) with synchronous peritoneal metastases (SPM) is controversial, and its prognosis remains poor. Here, we analysed the association between treatment strategies and the outcomes of patients with colorectal SPM and devised a nomogram to improve their prognosis prediction.

**Methods:**

We retrospectively analysed patients with colorectal SPM treated at The Sixth Affiliated Hospital, Sun Yat-sen University from June 2007 to June 2018. The Kaplan-Meier method with log-rank tests was used to compare the overall survival (OS) among patients undergoing different therapeutic regimens. Cox proportional hazards regression analysis was used to identify the prognostic factors. After variable selection, a nomogram was developed to predict the OS of patients with colorectal SPM.

**Results:**

A total of 371 patients with colorectal SPM were eligible for this study. The median OS of all patients was 15.0 months (95% CI, 13.1 to 16.9), with a 3-year and 5-year OS rate of 23.7% and 16.9%, respectively. Patients who underwent complete cytoreductive surgery (CC0–1) had a better median OS of 49 months (p<0.001). Cox multivariate analysis showed that age >65 years; cancer antigen 125 level >35 U/mL; peritoneal carcinomatosis index >16 scores; and undergoing cytoreductive surgery, chemotherapy and hyperthermic intraperitoneal chemotherapy were independent prognostic factors for OS. The c-index of the prognostic nomogram was 0.747 (95% CI, 0.474 to 1.020).

**Conclusions:**

Our study suggests that patients with colorectal SPM who receive comprehensive treatment might achieve better prognoses. The prognostic nomogram demonstrated good predictive performance for patients with colorectal SPM.

**WHAT IS ALREADY KNOWN ON THIS TOPIC**

- Current knowledge on the treatment and prognosis of colorectal cancer with synchronous peritoneal metastases is inconclusive, and a study with a large sample size from China is still lacking.

**WHAT THIS STUDY ADDS**

- This retrospective study from China suggested that undergoing cytoreductive surgery, chemotherapy and hyperthermic intraperitoneal chemotherapy were independent prognostic factors for patients with colorectal cancer with synchronous peritoneal metastases.
- Patients who received comprehensive treatment had a median overall survival of 29 months.

**HOW THIS STUDY MIGHT AFFECT RESEARCH, PRACTICE OR POLICY**

- This observational study indicated that patients with colorectal synchronous peritoneal metastases who received integrated treatment had better prognoses. Specifically, complete cytoreductive surgery should be considered to achieve long-term survival benefits. However, more refined stratification and randomised clinical trials are needed to address potential confounding factors and bias.

## Introduction

 Colorectal cancer (CRC) is the third most common malignant neoplasm worldwide, including in China.^1^,^3^ Nearly 25% of patients with CRC have metastatic disease at diagnosis.^4^ The typical locations for distant metastases are the liver, peritoneum and lung, among which synchronous peritoneal metastases (SPM) have worse progression-free survival and overall survival (OS).^5 6^ Early studies have shown that the median OS of patients with colorectal peritoneal metastases was ≤8 months; however, with the improvement of treatment strategies, the median OS has increased to 42 months.^7^

SPM are defined as peritoneal metastasis diagnosed at the same time as the primary tumour and are observed in 5%–10% of patients with CRC.^8 9^ The main treatments for SPM include cytoreductive surgery (CRS), systemic chemotherapy and hyperthermic intraperitoneal chemotherapy (HIPEC). However, the treatment of patients with colorectal SPM remains controversial in terms of the necessity of primary tumour resection, indications for CRS and HIPEC and regimens for perioperative chemotherapy.^10^,^12^

Initially, CRC with SPM was viewed as a terminal-stage disease for which palliative treatment was the only choice.^13^ In recent years, promising outcomes have been observed for patients undergoing selective CRS plus HIPEC.^14 15^ However, the National Comprehensive Cancer Network guideline seems conservative regarding CRS plus HIPEC and states that ‘The goal of treatment for most peritoneal metastases is palliative, rather than curative, and primarily consists of systemic therapy with palliative surgery or stenting if needed for obstruction or impending obstruction. CRS and/or intraperitoneal chemotherapy can be considered in experienced centres for selected patients with limited peritoneal metastases for whom R0 resection can be achieved.’ This guideline has limited indications for the clinical recommendation of CRS plus HIPEC, and clinicians still tend to favour palliative care for patients with CRC with peritoneal metastases. Hence, the optimal treatment approach for patients with colorectal SPM should be investigated further.

In addition, most studies on the treatment and prognosis of patients with colorectal SPM were from Europe and North America^416^,^18^ and a study with a large sample size from China is yet to be conducted. Hence, this study aimed to summarise the treatment strategies of patients with colorectal SPM and assess their clinical outcomes, as well as to develop a prognostic model to predict their OS.

## Patients and methods

### Patient information and inclusion criteria

In this observational retrospective study, the data of patients with colorectal SPM treated at The Sixth Affiliated Hospital, Sun Yat-sen University, Guangzhou, China, between June 2007 and June 2018 were retrieved. The following criteria were used to assess eligibility: (1) CRC with SPM confirmed by pathological diagnosis and (2) complete clinical information and follow-up. The exclusion criteria were as follows: (1) the presence of other malignant tumours or (2) death due to other diseases.

### Illustration of CRS, HIPEC and chemotherapy

Intraoperatively, the surgeons reassess the metastatic burden and use the peritoneal carcinomatosis index (PCI) to determine the feasibility of performing CRS, palliative resection or ostomy alone. Resection completeness was evaluated based on the completeness of cytoreduction score (CC): CC0, no macroscopic peritoneal tumour remained following cytoreduction; CC1, presence of tumour nodules <2.5 mm following cytoreduction; CC2, presence of residual disease measuring 2.5 mm to 2.5 cm; CC3, presence of tumour nodules >2.5 cm or a confluence of unresectable tumour nodules at any site within the abdomen or pelvis.^19^ For patients with obstruction caused by an unresectable tumour, intervention-guided stent placement, ostomy or bypass surgery was performed. HIPEC with 5-fluorouracil was usually performed on days 1–3 after CRS using the closed abdomen technique. In this study, chemotherapy means perioperative chemotherapy (neoadjuvant, adjuvant) or palliative chemotherapy. The chemotherapy regimen used was 5-fluorouracil-based chemotherapy (FOLFOX, FOLFIRI or XELOX). Targeted drugs, such as cetuximab or bevacizumab, were not considered in this study.

### Observations and variables

Based on the date of initial diagnosis, the data were classified into three 4-year periods: 2007–2010, 2011–2014 and 2015–2018. The patients were grouped based on the following three treatments: chemotherapy, HIPEC and CRS. Based on the number of treatments they received, the patients were allocated a score of 0, 1, 2 or 3 groups, indicating that they received none, 1, 2 or 3 of these three treatments, respectively.

The recorded data included age, sex, body mass index (BMI), tumour marker concentrations, location and stage of the primary tumour, pathological outcomes, perioperative chemotherapy, CRS and HIPEC. All patients were assessed preoperatively with a complete medical history and examination. Routine blood tests, biochemical tests, tumour marker assays and chest–abdomen–pelvic enhanced CT were performed. The surgeons decided whether to perform MRI based on tumour locations. Colonoscopy and biopsy were performed to confirm the preoperative pathological diagnosis. Tumour markers investigated included carcinoembryonic antigen (CEA), cancer antigen 19-9 (CA19-9) and cancer antigen 12-5 (CA12-5). The primary tumour locations were classified as the left side of the colon, right side of the colon, rectum or multiple sites. A multidisciplinary team reviewed all patients before surgery. PCI was assessed based on previous description by two experienced surgeons.^19^

### Outcomes and follow-up

The primary endpoint was OS, defined as the time from the date of initial treatment (chemotherapy or surgical intervention) to the date of death or last follow-up in censored patients. Survival status was determined via telephone follow-up or electronic medical records. Tumour marker assessment, CT and/or colonoscopy were performed at follow-up based on the patients’ condition and clinicians’ discretion.

### Statistical analysis

Statistical analysis was performed using SPSS software (V.25.0 for Windows; SPSS, Chicago, Illinois, USA) and R software (V.4.0.3). Descriptive statistics were used to analyse patients’ characteristics. Univariate and multivariate Cox proportional hazards regression analyses were used to identify variables related to OS. Variables with p<0.05 in univariate analysis were included in a multivariate analysis. OS was estimated using the Kaplan-Meier method and compared between groups using the log-rank test. Based on the results of multivariate analysis, a nomogram was established using R software. P value <0.05 was considered statistically significant.

## Results

### Patient characteristics

**Figure 1:**
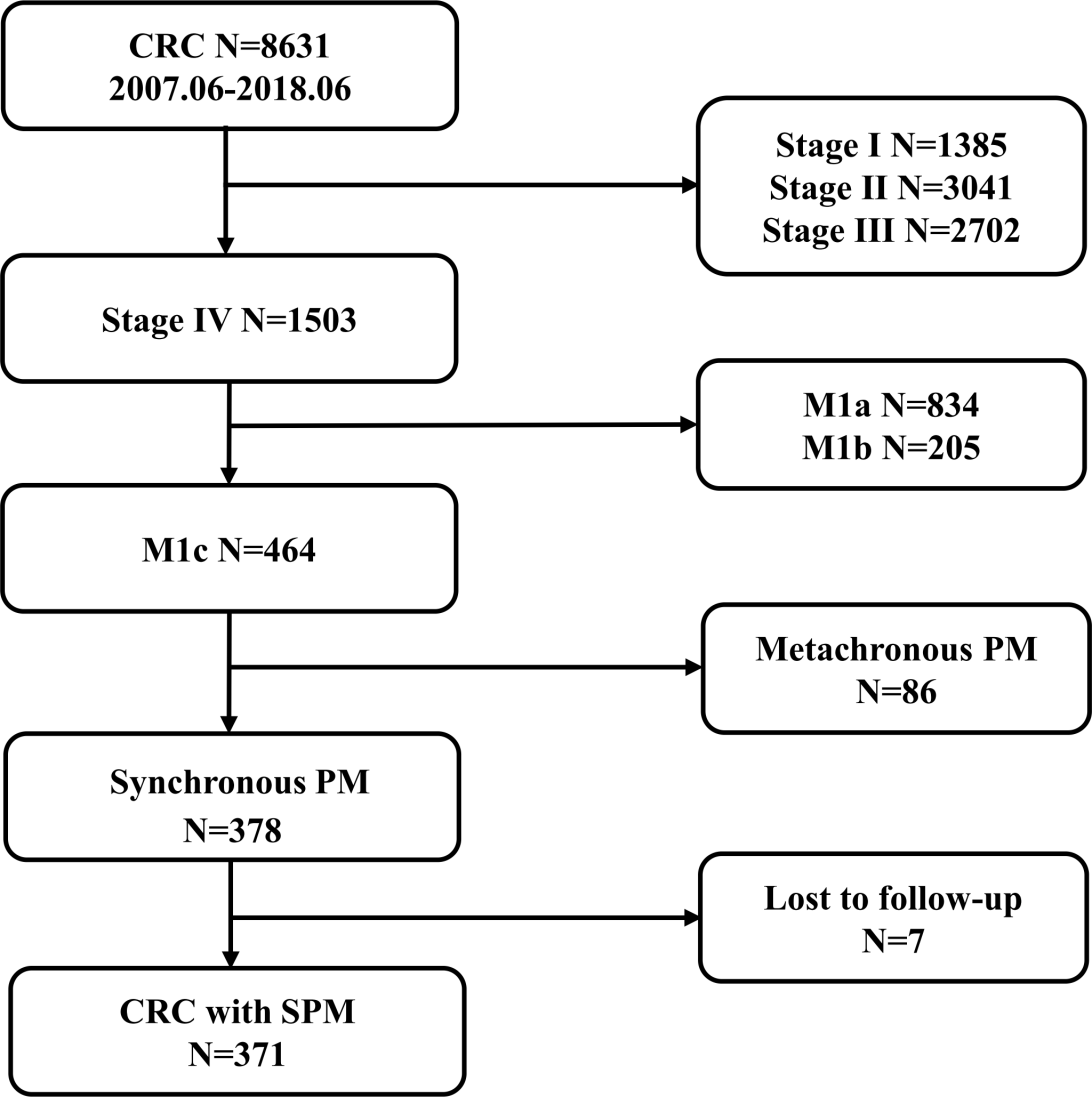
Study flowchart. Colorectal cancer with SPM in The Sixth Affiliated Hospital of Sun Yat-sen University from June 2007 to June 2018. CRC, colorectal cancer; PM, peritoneal metastasis; SPM, synchronous peritoneal metastases.

A total of 8631 patients with CRC were identified. Of these, 378 had SPM (4.4%; figure 1). Seven patients were excluded due to loss to follow-up or non-tumor-related death; finally, 371 patients were found eligible for this study.

Of the 371 patients, 95 (26%) were aged >65 years. The median age of the entire cohort was 58 (range: 18–92) years. The proportion of men was 63% (235/371). The preoperative diagnostic rate of peritoneal metastases by imaging (CT or MRI) was 53%. Furthermore, 133 (36%) patients had extraperitoneal metastasis, 226 (61%) had a complete or incomplete digestive obstruction and 240 (65%) had high or moderate histological differentiation (table 1).

**Table 1:** Characteristics of 371 patients with colorectal SPM

Variable	No. of cases (%)	Median OS (months)
Sex		
Male	235 (63)	14
Female	136 (37)	15
Age (years)		
≤65	276 (74)	17
>65	95 (26)	10
BMI (kg/m^2^)		
≤18.5	71 (19)	11
>18.5	300 (81)	15
CEA (ng/mL)		
≤10	191 (52)	15
>10	180 (48)	13
CA19-9 (U/mL)		
≤37	207 (56)	20
>37	164 (44)	11
CA12-5 (U/mL)		
≤35	146 (39)	24
>35	225 (61)	11
Imaging diagnosis		
No	175 (47)	16
Yes	196 (53)	13
Extraperitoneal metastasis		
No	238 (64)	16
Yes	133 (36)	12
Tumour location		
Right side	157 (42)	11
Left side	143 (39)	19
Rectum	64 (17)	12
Multisites	7 (2)	14
Tumour histology		
Adenocarcinoma	276 (75)	14
Mucinous adenocarcinoma	81 (22)	18
Signet ring cell carcinoma	14 (4)	15
Histological differentiation		
Low or undifferentiated	131 (35)	13
High or moderate	240 (65)	15
Digestive obstruction		
No	145 (39)	17
Complete	59 (16)	4
Incomplete	167 (45)	10
PCI		
≤16	248 (67)	20
>16	123 (33)	7
Ostomy or bypass		
No	292 (79)	18
Yes	79 (21)	7
CRS		
Not performed	116 (31)	6
CC0–1	87 (24)	49
CC2–3	168 (45)	13
Chemotherapy		
No	151 (41)	10
Yes	220 (59)	18
HIPEC		
No	265 (71)	12
Yes	106 (29)	24

BMI, body mass index; CA12-5, cancer antigen 12-5; CA19-9, cancer antigen 19-9; CC0, no macroscopic peritoneal tumour remains following cytoreductionCC1tumor nodules less than 2.5 mm persist following cytoreductionCC2residual disease measuring 2.5 mm to 2.5 cmCC3presence of tumor nodules greater than 2.5 cm, or a confluence of unresectable tumor nodules at any site within the abdomen or pelvisCEA, carcinoembryonic antigen; CRC, colorectal cancer; CRS, cytoreductive surgery; HIPEC, hyperthermic intraperitoneal chemotherapy; PCI, peritoneal carcinomatosis index; SPM, synchronous peritoneal metastases

### Treatment strategies

Among the 371 patients, 31 were diagnosed between 2007 and 2010, 101 between 2011 and 2014 and 239 between 2015 and 2018. Treatment regimens were assigned different scores according to the treatment method: score 0 (12%), score 1 (34%), score 2 (38%) and score 3 (16%) (table 2). The proportion of patients receiving HIPEC, systemic chemotherapy and comprehensive treatments increased markedly over time, whereas that of patients receiving CRS proportions did not change significantly. The proportion of patients receiving HIPEC increased from 0% to 36%, and that of patients who simultaneously received all three treatments increased from 0% to 21% from 2007 to 2018.

**Table 2:** Clinical information about treatment in three time periods

Periods	N	HIPEC, n (%)	CRS, n (%)	Chemotherapy, n (%)	Score 0, n (%)	Score 1, n (%)	Score 2, n (%)	Score 3, n (%)
Entire cohort	371	106 (29)	255 (69)	220 (59)	44 (12)	127 (34)	142 (38)	58 (16)
2007–2010	31	0 (0)	22 (71)	13 (42)	5 (16.1)	16 (52)	10 (32)	0 (0)
2011–2014	101	20 (20)	74 (73)	45 (45)	15 (15)	41 (41)	36 (36)	9 (9)
2015–2018	239	86 (36)	159 (67)	162 (68)	24 (10)	70 (29)	96 (40)	49 (21)

Score 0, not receiving of HIPEC, CRS or systemic chemotherapy. Score 1, receiving one of the three types of treatment. Score 2, receiving two of the three types of treatment. Score 3, receiving all the three types of treatment.

CRScytoreductive surgeryHIPEC, hyperthermic intraperitoneal chemotherapyN, number

### Prognostic factors affecting OS

On univariate analysis, age >65 years (p<0.001), extraperitoneal metastasis (p=0.006), CA19−9 >37 U/mL (p<0.001), CA12−5 >35 U/mL (p<0.001) and PCI >16 (p<0.001) were significantly associated with poorer OS, whereas CC0–1 (p<0.001), CC2–3 (p<0.001), receiving chemotherapy (p=0.001), receiving HIPEC (p<0.001) and tumour located in the left side (p=0.002) were significantly associated with improved OS. Multivariate analysis showed that age >65 years (p=0.001), CA12−5 >35 U/mL (p=0.004), PCI >16 (p=0.009), CC0–1 (p<0.001), CC2–3 (p=0.013), receiving chemotherapy (p=0.003) and receiving HIPEC (p=0.002) were independent prognostic factors for OS (table 3).

**Table 3:** Univariate and multivariate analyses for overall survival of 371 patients with colorectal SPM using the Cox proportional hazards regression

Variable	Univariate analysis		Multivariate analysis	
	HR (95% CI)	P value	HR (95% CI)	P value
Sex (female)	0.899 (0.704 to 1.147)	0.390		
Age (>65 years)	1.841 (1.426 to 2.378)	<0.001	1.573 (1.207 to 2.049)	0.001
CEA (>10 ng/mL)	1.060 (0.836 to 1.343)	0.630		
CA19-9 (>37 U/mL)	1.756 (1.388 to 2.222)	<0.001	1.231 (0.927 to 1.584)	0.106
CA12-5 (>35 U/mL)	1.844 (1.442 to 2.360)	<0.001	1.476 (1.130 to 1.926)	0.004
Digestive obstruction (yes)	1.161 (0.912 to 1.476)	0.225		
Tumour location				
Right side	Reference			
Left side	0.653 (0.502 to 0.851)	0.002		
Rectum	0.797 (0.573 to 1.109)	0.179		
Multisides	1.333 (0.622 to 2.855)	0.460		
Extraperitoneal metastasis (yes)	1.403 (1.104 to 1.783)	0.006	1.118 (0.870 to 1.437)	0.383
Tumour histology				
Adenocarcinoma	Reference			
Mucinous adenocarcinoma	0.927 (0.699 to 1.229)	0.598		
Signet ring cell carcinoma	1.191 (0.649 to 2.186)	0.573		
Histological differentiation				
Low or undifferentiated	Reference			
High or moderated	0.806 (0.632 to 1.028)	0.082		
PCI (>16)	2.546 (1.990 to 3.257)	<0.001	1.445 (1.096 to 1.907)	0.009
CRS				
Not performed	Reference			
CC0–1	0.176 (0.121 to 0.255)	<0.001	0.275 (0.181 to 0.417)	<0.001
CC2–3	0.587 (0.454 to 0.760)	<0.001	0.706 (0.536 to 0.928)	0.013
Chemotherapy (yes)	0.679 (0.536 to 0.860)	0.001	0.687 (0.538 to 0.878)	0.003
HIPEC (yes)	0.552 (0.417 to 0.730)	< 0.001	0.630 (0.473 to 0.840	0.002

CA12-5, cancer antigen 12-5; CA19-9, cancer antigen 19-9; CC0no macroscopic peritoneal tumour remains following cytoreductionCC1tumor nodules less than 2.5 mm persist following cytoreductionCC2residual disease measuring 2.5 mm to 2.5 cmCC3presence of tumor nodules greater than 2.5 cm or a confluence of unresectable tumor nodules at any site within the abdomen or pelvisCEA, carcinoembryonic antigen; CRC, colorectal cancer; CRS, cytoreductive surgery; HIPEChypothermic intraperitoneal chemotherapyPCI, peritoneal carcinomatosis index; SPM, synchronous peritoneal metastases

### Survival information

**Figure 2:**
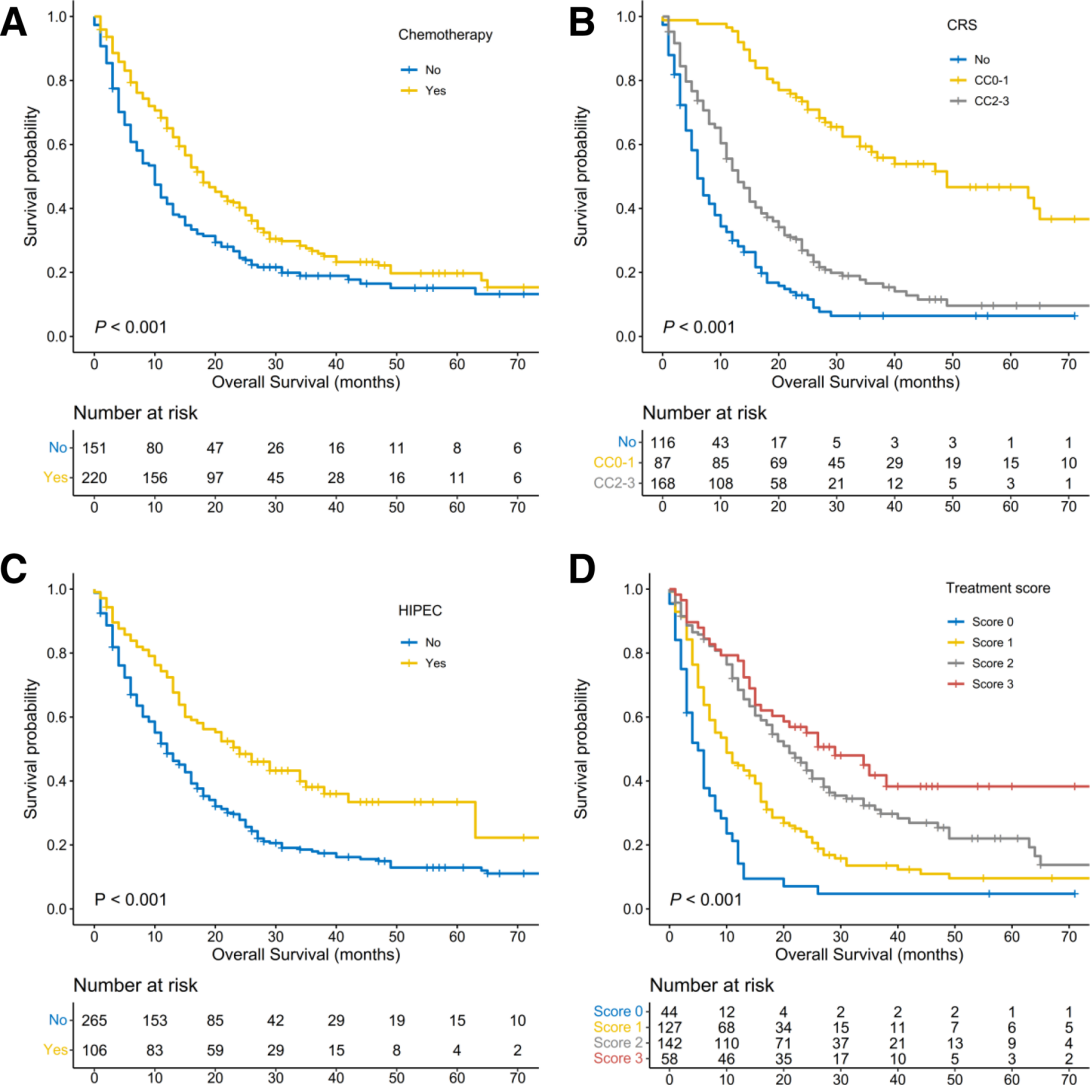
Overall survival of patients with colorectal synchronous peritoneal metastases. (A) Overall survival stratified by chemotherapy (p<0.001). (B) Overall survival stratified by CRS (p<0.001). (C) Overall survival stratified by HIPEC (p<0.001). (D) Overall survival stratified by treatment scores (p<0.001). CC0, no macroscopic peritoneal tumour remains following cytoreduction; CC1, tumor nodules less than 2.5 mm persist following cytoreduction; CC2, residual disease measuring 2.5 mm to 2.5 cm; CC3, presence of tumor nodules greater than 2.5 cm, or a confluence of unresectable tumor nodules at any site within the abdomen or pelvis; CRS, cytoreductive surgery; HIPEC, hypothermic intraperitoneal chemotherapy.

The median follow-up time was 44.0 months (95% CI, 33.6 to 54.4) for the entire population, and 282 patients (76.0%) died during follow-up. The median OS was 15.0 months (95% CI, 13.1.0 to 16.9) in the entire population, and the 3-year and 5-year OS rates were 23.7% and 16.9%, respectively. Kaplan-Meier analysis showed that undergoing chemotherapy (p<0.001), CRS (p<0.001) and HIPEC (p<0.001) were significantly associated with improved OS. Stratified by chemotherapy, the median OS was 18 months in the chemotherapy group and 10 months in the other group. Stratified by the CRS, the median OS of patients who did not receive CRS was 6 months, while that of patients who received CRS of CC0–1 and CC2–3 was 49 and 13 months, respectively. HIPEC was significantly associated with increased OS, with a median OS of 24 months in the HIPEC group and 12 months in the non-HIPEC group (figure 2). The median OS of patients with treatment scores of 0, 1, 2 and 3 was 5, 10, 21 and 29 months, respectively, with 3-year survival rates of 0%, 12.3%, 31.0% and 41.8%, respectively.

### Establishment and assessment of the nomogram for prognosis prediction

**Figure 3:**
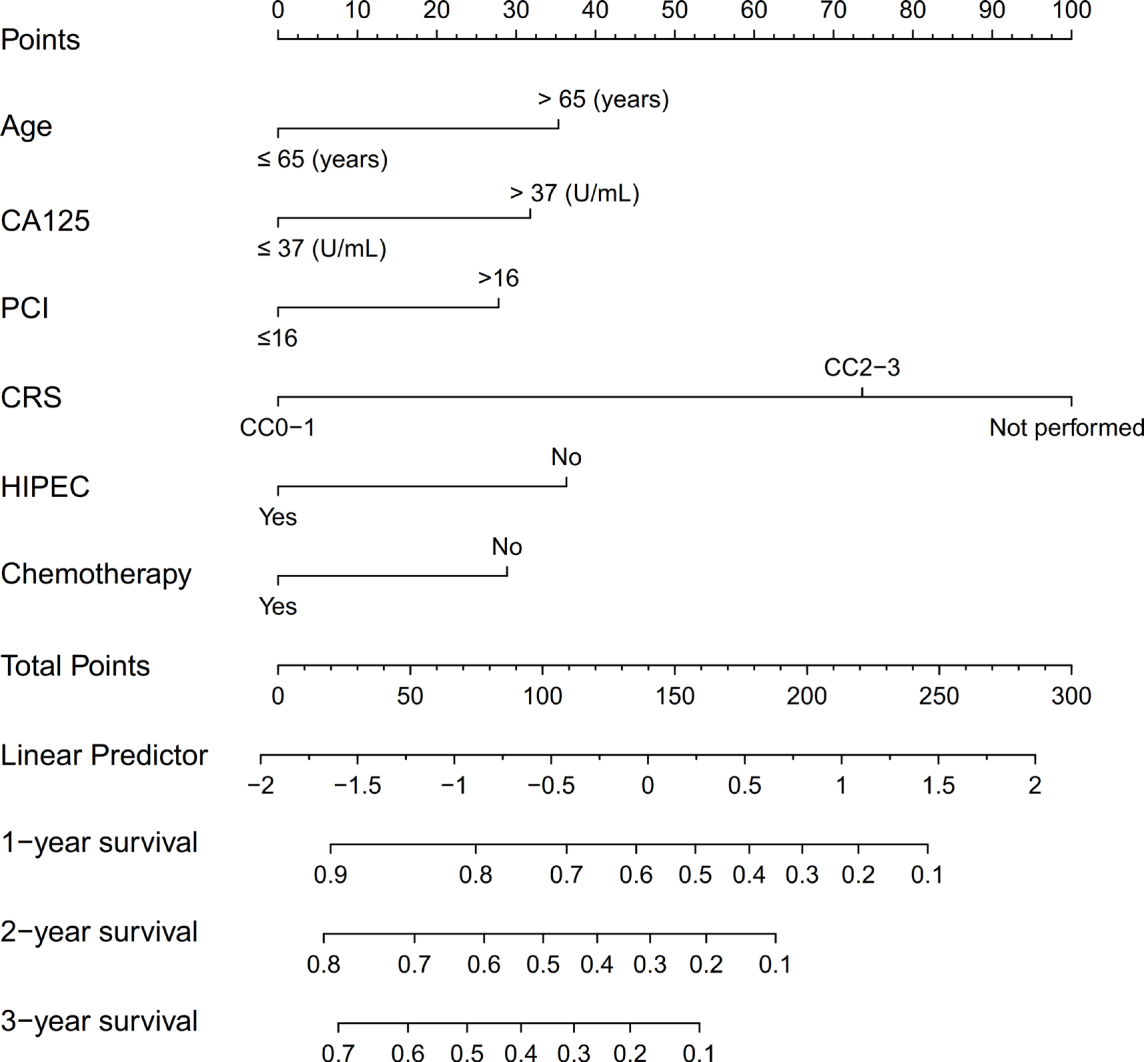
A nomogram model for predicting overall survival in patients with colorectal synchronous peritoneal metastases (a dynamic nomogram for patients with colorectal cancer with synchronous peritoneal metastases at https://qxssysu.shinyapps.io/CRCSPM_OS_DynNomapp). CA12-5, cancer antigen 12-5; CRS, cytoreductive surgery; HIPEC, hypothermic intraperitoneal chemotherapy; PCI, peritoneal carcinomatosis index.

**Figure 4:**
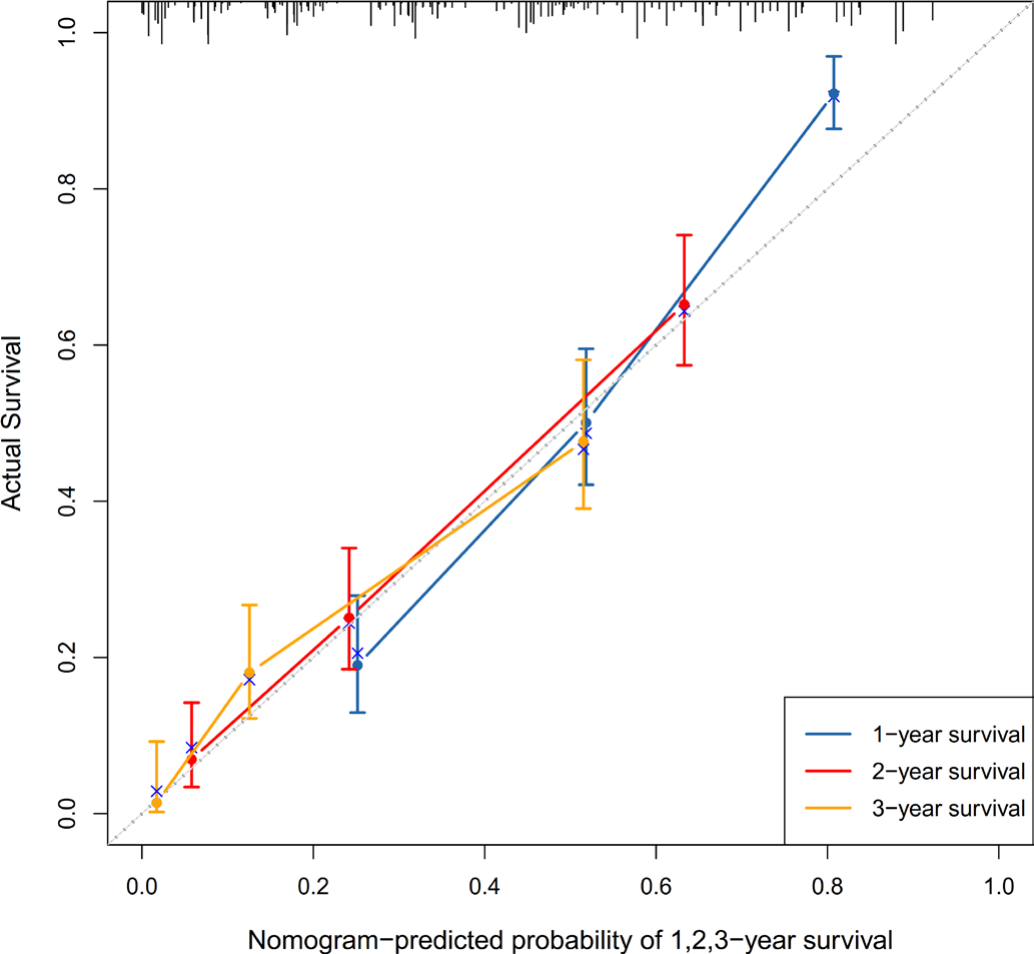
The calibration curves for validation of the nomogram. Predicted 1-year, 2-year and 3-year overall survivals for patients with colorectal synchronous peritoneal metastases are shown on the horizontal axis, and actual 1-year, 2-year and 3-year overall survivals are shown on the vertical axis. The c-index is 0.747 (95% CI, 0.474 to 1.020).

Variables with p<0.05 from the multivariate analysis were used to establish a nomogram for predicting prognosis (figure 3). This model could predict the 1-year, 2-year and 3-year OS rates of patients with SPM. Then, the performance of the nomogram was verified using the c-index, calibration curve and receiver operating characteristic (ROC) curve. The c-index of the model was 0.747 (95% CI, 0.474 to 1.020). The c-index, calibration and ROC curves indicated satisfactory prediction performance (figures4  5). In order to increase practicality, we further constructed a dynamic nomogram, which can be found at https://qxssysu.shinyapps.io/CRCSPM_OS_DynNomapp.

**Figure 5:**
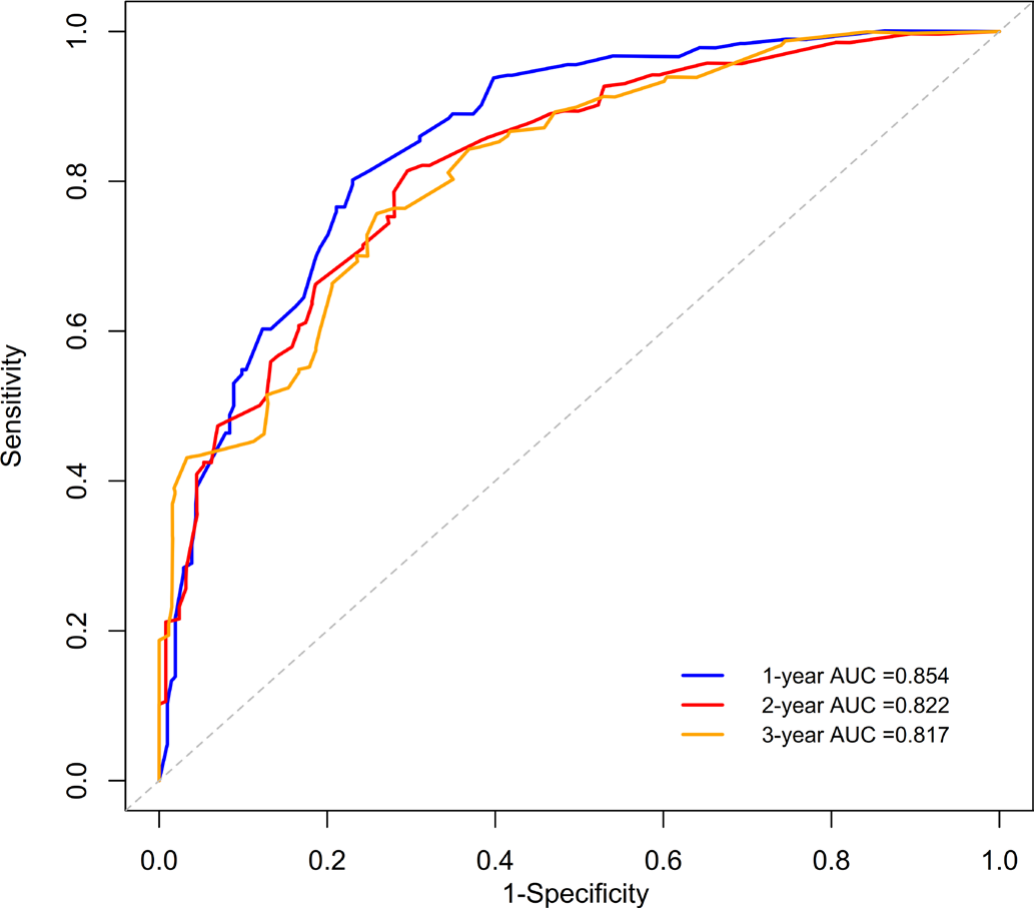
Receiver operating characteristic (ROC) curves of the nomogram for predicting 1-year, 2-year and 3-year overall survival of patients with colorectal synchronous peritoneal metastases. AUC, area under the curve.

## Discussion

Patients with colorectal SPM have a poor prognosis, and the eighth edition of the American Joint Committee on Cancer (AJCC) staging system classifies peritoneal1 metastases as M1c. In this single-centre study, we identified the prognostic factors associated with OS. Our findings suggest that patients with colorectal SPM who received comprehensive treatment achieved better prognoses and complete CRS should be considered for long-term survival benefits in patients with SPM of colorectal origin.

Previous studies from China have investigated the association between treatment strategies and OS in patients with colorectal SPM.^20^,^23^ However, their clinical significance was limited due to the small number of patients investigated,^20^,^23^ involving metachronous peritoneal metastases,^20^ involving appendiceal tumours^22^ or patients being treated only CRS plus HIPEC.^23^ Although numerous international multicentre cohort studies have been conducted on patients with colorectal SPM, they mainly focused on the benefits of CRS plus HIPEC or primary tumour resection.^10 11 24 25^ Compared with these studies, the conclusions of the present study are more general.

The prevalence of SPM was 4.4% in this study, similar to that reported in most previous studies.^4 24 26^ Our centre is one of the largest gastrointestinal treatment centres in South China. Thus, our data may be representative to some extent. In this study, HIPEC has been increasingly used, reflecting the acceptance of the CRS plus HIPEC treatment pattern by surgeons. Of note, between 2015 and 2018, 41% of patients with SPM received only one type of treatment, mainly treated by ostomy or systemic chemotherapy. In these patients, the volume of SPM might be too large to perform CRS. Another possible reason is that CRS was not widely accepted in the past years. Currently, most surgeons still resort to conservative treatment for SPM.

Our study showed that CRS, HIPEC and systemic chemotherapy are independently associated with OS. Patients who received all three treatments had a median OS of 29 months, which was longer than those receiving any one or two modalities of treatments. Previous studies showed that patients who received CRS plus HIPEC had a median OS of 32.4–62.7 months,^1627^,^30^ which was longer than that observed in the present study. This might be because most patients who received CRS in our study did not undergo complete cytoreduction. However, patients who received CRS of CC0–1 had a median OS of 49 months, which was consistent with the findings reported in the PRODIGE 7 trail.^7^ Patients with SPM generally have an inferior status,^31^ and the morbidity and mortality rates of CRS plus HIPEC are 20%–40% and 3%, respectively.^32^ Therefore, the management of these patients requires the collaborative efforts of radiologists, medical oncologists, radiation oncologists and surgeons from several disciplines.

The prognostic nomogram showed moderate predictive ability, which can aid clinical decision making. Previous studies have proposed models for predicting the prognosis of CRC with peritoneal metastases. The most commonly mentioned scores are the Peritoneal Surface Disease Severity Score (PSDSS) and Colorectal Peritoneal Metastases Prognostic Surgical Score (COMPASS).^33 34^ PSDSS is used to predict the prognosis of patients with peritoneal metastases after CRS plus HIPEC in combination with clinical symptoms, CT-PCI and histopathological characteristics of the primary tumour. However, this scoring system is mainly used to select patients suitable for CRS plus HIPEC, and its clinical application is relatively limited. COMPASS further optimised the indices of PSDSS and visualised the prediction model without changing the application premise, and the c-index of the model was 0.720. Compared with PSDSS and COMPASS, the nomogram established in this study can be more widely used and has a better prediction ability, with a c-index of 0.747.

The poor prognosis of SPM emphasises the importance of early diagnosis. Unfortunately, imaging examinations are limited in detecting minor lesions.^35^ In our study, only 52.8% of patients with SPM were diagnosed using preoperative imaging examinations. Although positron emission tomography (PET)-CT has a higher detection rate, clinicians do not use it routinely because of its high cost. Therefore, it is important to accurately identify these high-risk patients and perform routine PET-CT. In addition, new techniques can also be considered for the early diagnosis of PM. Dong *et al* developed a radiomic nomogram to identify occult PM in patients with advanced gastric cancer, and the area under the curve (AUC) was 0.920 in the external validation cohort.^36^ We recently reported an image-based deep learning algorithm to identify SPM of colorectal origin based on CT images, and it has shown great potential in the prediction of SPM, showing an accuracy of 94.11% with an AUC 0.922.^37^ Furthermore, studies in recent years have indicated that circulating tumour DNA is of great value in detecting minimal residual disease and monitoring the recurrence and treatment response of CRC.^38^,^40^ Circulating tumour DNA from peritoneal lavage fluids may be helpful to identify patients at high risk of metachronous peritoneal metastases.

This study has some limitations. First, as this was a retrospective study, we relied on the accuracy of pre-input data. To be as accurate as possible, two doctors checked all the data. Second, in this study, chemotherapy was defined as receiving at least two consecutive courses of systemic chemotherapy during perioperative or palliative care, and a specific chemotherapy regimen; the application of targeted or immunological drugs and the course of chemotherapy were not subdivided, which could lead to some inherent potential bias. Finally, this study lacks validation, and the Surveillance, Epidemiology, and End Results database should be considered for validation in the future.

## Conclusions

This observational study indicated that patients with colorectal SPM who received integrated treatment had better prognoses and that complete CRS should be considered for long-term survival benefits. The developed nomogram demonstrated good predictive power, which could be used for the prognostic assessment of patients with colorectal SPM and for selecting patients suitable for CRS plus HIPEC. Certainly, more refined stratification and randomised clinical trials are needed to address potential confounding factors and bias.

## supplementary material

10.1136/egastro-2023-100016 Uncited online supplemental file 1

## Data Availability

Data are available upon reasonable request.
