# Supplementary material for: Treatment and prognosis of colorectal cancer with synchronous peritoneal metastases: 11-year single institute experience
Source: eGastroenterology. 2023 Sep 20;1(2):e100016. doi: 10.1136/egastro-2023-100016 (PMC11741188; doi:10.1136/egastro-2023-100016)
Supplement: Uncited online supplemental file 1 [file egastro-1-2-s001.pptx]

## Slide 1
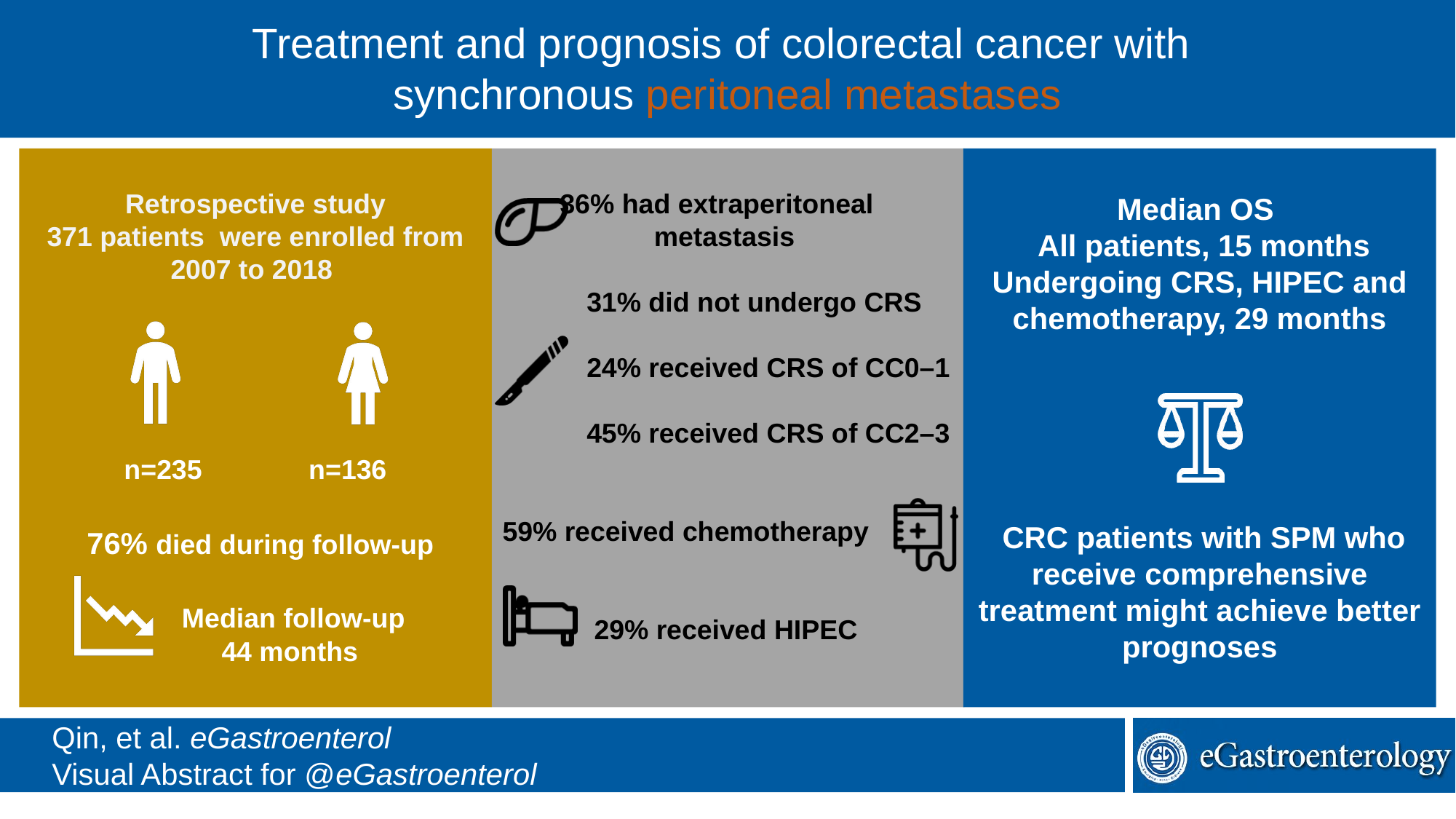

# Treatment and prognosis of colorectal cancer with synchronous peritoneal metastases
Retrospective study
371 patients were enrolled from 2007 to 2018
n=235 n=136
 76% died during follow-up
 Median follow-up
 44 months
 36% had extraperitoneal metastasis
 31% did not undergo CRS
 24% received CRS of CC0–1
 45% received CRS of CC2–3
59% received chemotherapy
 29% received HIPEC
Median OS
 All patients, 15 months
Undergoing CRS, HIPEC and chemotherapy, 29 months
 CRC patients with SPM who receive comprehensive treatment might achieve better prognoses
 Qin, et al. eGastroenterol
 Visual Abstract for @eGastroenterol
