# Supplementary material for: Treatment and prognosis of colorectal cancer with synchronous peritoneal metastases: 11-year single institute experience
Source: eGastroenterology. 2023 Sep 20;1(2):e100016. doi: 10.1136/egastro-2023-100016 (PMC11741188; doi:10.1136/egastro-2023-100016)
Supplement: Supplementary file 2 [file egastro-2023-100016.reviewer_comments.pdf]

PEER REVIEW HISTORY

ARTICLE DETAILS

TITLE(PROVISIONAL)	Treatment and prognosis of colorectal cancer with synchronous peritoneal metastases: 11-year single institute experience
AUTHORS	Qin, Xiusen; Yang, Zifeng; Li, Yang; Luo, Jian; Wang, Hui; Wang, Huaiming

VERSION 1 – REVIEW

REVIEWER 1	Harmon, John<br>Johns Hopkins University, USA
REVIEW RETURNED	02-Nov-2022

GENERAL COMMENTS	Number EG202002<br><br>Treatment and prognosis of colorectal cancer with synchronous peritoneal metastases: 12-year single institute experience<br><br>Quinn at all<br><br>Introduction<br><br>This paper describes the experience of the Sun Yat-sen 6th Affiliated Hospital caring for patients with colorectal cancer and synchronous peritoneal metastases. They identify factors that independently predict prognosis either positively or negatively. Therapeutic options of chemotherapy, HIPEC, and cytoreductive surgery were all found to be independent predictors of improved survival.<br><br>Then they created a nomogram for predicting survival for these patients.<br><br>Critique<br><br>Overall the paper is very interesting and well written, but some issues need to be clarified.<br><br>The treatment groups need clarification. It is very confusing as presented in the abstract. What is the significance of multiple treatments with one modality. Does that increase the index number? And are all 3 treatment modalities really equal? They are clearly not based on the results of single treatments. Is this a

	problem? At the least the explanation of the numerical system needs to be clearly explained. It might be best to leave that out of the abstract because it is very complicated. The explanations in the text are more easily understood<br><br>Also, the PCI needs to be described and referenced. The protocol needs to be briefly described. The use of the PCI by the surgeons performing cytoreduction for this group of patients needs to be discussed. What is their upper limit for performing cyto-reductive surgery? Were the other therapeutic strategies used when that number was exceeded?<br><br>The systemic chemo therapy regimens using 5FU are described. Did patients also receive immuno therapy? The paper says (line 104) that immunotherapy is "not discussed". But was it administered to some of the patients?<br><br><br>Minor<br>1. Please provide a list identifying all the abbreviations.<br>2. The percentages are presented as tenths. Please change them to full numerals. (11% not 10.7%)

REVIEWER 2	Wlodarczk , Jakub<br>Johns Hopkins Medicine, USA
REVIEW RETURNED	02-Nov-2022

GENERAL COMMENTS	This paper, entitled "Treatment and prognosis of colorectal cancer with synchronous peritoneal metastases: 12-year single institute experience" provides valuable results regarding different and combined approaches in treating the synchronous peritoneal metastases in CRC. The paper is interesting and the matter of your study is highly relevant.<br><br>I suggest revisions:<br>1. In lines 57-60 authors state that there is no formal consensus regarding standardized criteria. That is not completely true. Please mention following papers in the introduction or discussion:<br><br>Klaver, C.E.L., Groenen, H., Morton, D.G., Laurberg, S., Bemelman, W.A., Tanis, P.J. and (2017), Recommendations and consensus on the treatment of peritoneal metastases of colorectal origin: a systematic review of national and international guidelines. Colorectal Dis, 19: 224-236. https://doi.org/10.1111/codi.13593

Adamina, M.; Warlaumont, M.; Berger, M.D.; Däster, S.; Delaloye, R.; Digklia, A.; Gloor, B.; Fritsch, R.; Koeberle, D.; Koessler, T.; Lehmann, K.; Müller, P.; Peterli, R.; Ris, F.; Steffen, T.; Weisshaupt, C.S.; Hübner, M. Comprehensive Treatment Algorithms of the Swiss Peritoneal Cancer Group for Peritoneal Cancer of Gastrointestinal Origin. Cancers 2022, 14, 4275. https://doi.org/10.3390/cancers14174275

2. In lines 68-70 authors mention that CRS and/or intraperitoneal chemotherapy can be considered …for patients for whom R0 resection can be achieved. However, in your paper you don't provide any results regarding the resection of primary tumor and the R0 resection rate.

3. Lines 98-99: "For patients with obstruction caused by an unresectable tumor, only intervention-guided stent placement or ostomy was performed."

Were those patients included into final analysis? Please provide specific numbers for patients each treated with stent or ostomy. Did they receive palliative chemotherapy?

4. Lines 145-146: "Further, 133 (35.8%) had extraperitoneal metastasis, and 226 (60.9%) had a complete or incomplete digestive obstruction…"

Please distinguish and provide numbers for complete and incomplete digestive obstruction. Moreover, authors mention that only 21.3% of patients had ostomy or bypass. Please mention how other patients with obstruction were treated.

5. There are no results about the number of resected lymph nodes and metastases to lymph nodes in operated patients.

Please provide these results.

Minor comments:

1. Line 75-6: Writing typos "strategies for CRC with SPM…model to s// better…"

2. Lines 144-145: "The diagnosis rate by imaging examination (CT or MRI) was 52.8%"

I assume that the rest of cases were diagnosed intraoperatively? Please make it clear in the paper. Was this for the diagnosis of peritoneal metastases.?

3. Lines 240-242: Authors mention about image-based deep learning algorithm to identify lesions with SPM for CRC and its great potential in the prediction of SPM. Please provide more specific information

4. Lines 242-245: Authors suggest that circulating tumor DNA (ctDNA), which are used in monitoring minimal residual disease

	(MRD), may be helpful in identifying the potential peritoneal metastases. Since minimal residual disease and peritoneal metastases are not the same, please provide more rationale to sustain your suggestion.<br>5. Lines 159-160: Please provide an explanation for CCR abbreviation.

VERSION 1 – AUTHOR RESPONSE

Reviewer 1:

General Comment: This paper describes the experience of the Sun Yat-sen 6th Affiliated Hospital caring for patients with colorectal cancer and synchronous peritoneal metastases. They identify factors that independently predict prognosis either positively or negatively. Therapeutic options of chemotherapy, HIPEC, and cytoreductive surgery were all found to be independent predictors of improved survival. Then they created a nomogram for predicting survival for these patients. Overall the paper is very interesting and well written, but some issues need to be clarified.
Response: Thank you very much for taking the time to review this manuscript. According to the reviewer's suggestions, we have revised our manuscript.

Comment 1: The treatment groups need clarification. It is very confusing as presented in the abstract. What is the significance of multiple treatments with one modality. Does that increase the index number? And are all 3 treatment modalities really equal? They are clearly not based on the results of single treatments. Is this a problem? At the least the explanation of the numerical system needs to be clearly explained. It might be best to leave that out of the abstract because it is very complicated. The explanations in the text are more easily understood.
Response: Thank you for your valuable comments. We have modified the methods section of the abstract to make it easier to understand.

Comment 2: Also, the PCI needs to be described and referenced. The protocol needs to be briefly described. The use of the PCI by the surgeons performing cytoreduction for this group of patients needs to be discussed. What is their upper limit for performing cyto-reductive surgery? Were the other therapeutic strategies used when that number was exceeded?
Response: Thank you for your advice. PCI was first proposed by Sugarbaker in 1996, and due to its relatively complex description, we cite its sources (Line 122, Ref 19).
To date, there is no standard upper PCI score cutoff to consider CRS. However, early work suggested using a PCI score cutoff of less than 20 to determine which patients may benefit from CRS (J Clin Oncol. 2009 Feb 10;27(5):681-5.). This proposal was then refined to suggest that patients with a PCI score of less than 12 should be considered for resection and that patients with a PCI score of greater than 17 are unlikely to benefit from cytoreduction (Surg Oncol Clin N Am. 2018 Jul;27(3):563-583.). In this study, the cut-off value is 16. For patients

with a high PCI score, the decision to proceed with cytoreduction should be taken inlight of the patient's overall health, the tumor biology, and the ability to achieve a complete resection. Some patients with a high PCI score may be suitable for palliative treatment.

Comment 3: The systemic chemotherapy regimens using 5FU are described. Did patients also receive immunotherapy? The paper says (line 104) that immunotherapy is "not discussed". But was it administered to some of the patients?
Response: Thank you for your comments. The paper says (line 104) that "Targeted drugs, such as Cetuximab or Bevacizumab, were not discussed in this study".
Patients included in this study were before June 2018, and the immunotherapy drugs were launched late in China. Hence, only a very small number of patients received immunotherapy in this study. However, 20% of patients received treatments such as Cetuximab or Bevacizumab in addition to systemic chemotherapy. Peritoneal metastases were advanced disease, and chemotherapy regimens often change as the disease changes. In order to reduce confounding factors, the use of Cetuximab, Bevacizumab and immunodrugs were not considered in this study.

Comment 4: Please provide a list identifying all the abbreviations.
Response: Thank you for your advice. There are a lot of abbreviations in the manuscript, and we have added a list of abbreviations at the end of the manuscript.

Comment 5: The percentages are presented as tenths. Please change them to full numerals. (11% not 10.7%)
Response: Thank you for your advice. We have modified them as required.

Reviewer: 2

General Comment: This paper, entitled "Treatment and prognosis of colorectal cancer with synchronous peritoneal metastases: 12-year single institute experience" provides valuable results regarding different and combined approaches in treating the synchronous peritoneal metastases in CRC. The paper is interesting and the matter of your study is highly relevant.
Response: Thank you for your valuable comments.

Comment 1: In lines 57-60 authors state that there is no formal consensus regarding standardized criteria. That is not completely true. Please mention following papers in the introduction or discussion:

Klaver, C.E.L., Groenen, H., Morton, D.G., Laurberg, S., Bemelman, W.A., Tanis, P.J. and (2017), Recommendations and consensus on the treatment of peritoneal metastases of colorectal origin: a systematic review of national and international guidelines. Colorectal Dis, 19: 224-236. https://doi.org/10.1111/codi.13593

Adamina, M.; Warlaumont, M.; Berger, M.D.; Däster, S.; Delaloye, R.; Digklia, A.; Gloor, B.;

Fritsch, R.; Koeberle, D.; Koessler, T.; Lehmann, K.; Müller, P.; Peterli, R.; Ris, F.; Steffen, T.; Weisshaupt, C.S.; Hübner, M. Comprehensive Treatment Algorithms of the Swiss Peritoneal Cancer Group for Peritoneal Cancer of Gastrointestinal Origin. Cancers 2022, 14, 4275. https://doi.org/10.3390/cancers14174275

Response: Thank you for your valuable comments. The original expression is not appropriate, we have revised the expression of this part. The revised statement is as follows:

However, the treatment of CRC patients with SPM remains controversial in terms of the necessity of the primary tumor resection, indications of CRS and HIPEC, and regimens for perioperative chemotherapy.

Comment 2: In lines 68-70 authors mention that CRS and/or intraperitoneal chemotherapy can be considered …for patients for whom R0 resection can be achieved. However, in your paper you don't provide any results regarding the resection of primary tumor and the R0 resection rate.

Response: Thank you for your comments. In the present study, according to the CC score, completeness of cytoreduction is divided into not undergoing cytoreductive surgery (CRS), CC0-1 and CC2-3. All patients with CC score (CC1-3) underwent primary tumor resection. CC0 patients are equivalent to R0 resection. However, according to the guidelines of the International Peritoneal Cancer Society, CC0-1 is considered to achieve complete CRS and patients can continue to receive HIPEC. Therefore, patients with CC0 and CC1 were analyzed together in this study.

Comment 3: Lines 98-99: "For patients with obstruction caused by an unresectable tumor, only intervention-guided stent placement or ostomy was performed." Were those patients included into final analysis? Please provide specific numbers for patients each treated with stent or ostomy. Did they receive palliative chemotherapy?

Response: Thank you for your careful review. First of all, the sentence you mentioned is not an accurate statement in the manuscript. We have modified it as "For patients with obstruction caused by an unresectable tumor, intervention-guided stent placement, ostomy or bypass surgery was performed." Secondly, these patients were also included in the study and some of them (58%) received palliative chemotherapy. The rest may forgo treatment for financial reasons. They were classified into not undergoing CRS group. Thirdly, the specific number for stent placement, ostomy, and bypass was 15, 69, and 10, respectively. Besides, not undergoing CRS group (116 patients) also included 22 patients underwent an "on-off" surgery.

Comment 4: Lines 145-146: "Further, 133 (35.8%) had extraperitoneal metastasis, and 226 (60.9%) had a complete or incomplete digestive obstruction…"

Please distinguish and provide numbers for complete and incomplete digestive obstruction. Moreover, authors mention that only 21.3% of patients had ostomy or bypass. Please mention how other patients with obstruction were treated.

Response: Thank you for your comments. It is important to distinguish between complete and incomplete digestive obstruction. At your suggestion, we distinguished between complete (59/371 patients) and incomplete (167/371patients) ileus and supplement this data in the

baseline table. 21.3% (79/371) patients received ostomy or bypass. 15 patients with obstruction caused by an unresectable tumor received stent placement. Other patients with obstruction received CRS.

Comment 5: There are no results about the number of resected lymph nodes and metastases to lymph nodes in operated patients. Please provide these results.
Response: Thank you for your comments. The effect of the number of resected lymph nodes removed on prognosis was not analyzed in the present study. Moreover, most literatures only showed lymph node staging or whether they existed metastasis (Lancet Oncol. 2021 Feb;22(2):256-266; JAMA Oncol. 2020 Aug 1;6(8):e202701). In view of the concern you mentioned, we reviewed the postoperative pathological reports of these patients. The median (IQR) of the number of resected lymph nodes is 20 (15-24).

Comment 6: Line 75-6: Writing typos "strategies for CRC with SPM…model to s// better…"
Response: Thank you for your careful review. We have corrected this mistake.

Comment 7: Lines 144-145: "The diagnosis rate by imaging examination (CT or MRI) was 52.8%". I assume that the rest of cases were diagnosed intraoperatively? Please make it clear in the paper. Was this for the diagnosis of peritoneal metastases.?
Response: Thank you for your careful review. As you assumed, 52.8% refers to the diagnosis of peritoneal metastasis on preoperative imaging examination (CT or MRI). The rest of patients were diagnosed with peritoneal metastases intraoperatively. Most patients with peritoneal metastases were not clearly diagnosed before surgery, which may be related to the experience of imaging diagnostic doctors. On the other hand, some peritoneal metastases themselves were relatively occult. This also suggests the importance of multidisciplinary teams in the diagnosis and treatment of cancer

Comment 8: Lines 240-242: Authors mention about image-based deep learning algorithm to identify lesions with SPM for CRC and its great potential in the prediction of SPM. Please provide more specific information.
Response: Thank you for your comments. We have provided more detailed information as requested as follows:
"… and it has shown great potential in the prediction of SPM, which showed accuracy of 94.11% with AUC of 0.922."

Comment 9: Lines 242-245: Authors suggest that circulating tumor DNA (ctDNA), which are used in monitoring minimal residual disease (MRD), may be helpful in identifying the potential peritoneal metastases. Since minimal residual disease and peritoneal metastases are not the same, please provide more rationale to sustain your suggestion.
Response: Thank you for your valuable comments. We have modified the content of this part as follows:
"ctDNA from peritoneal lavage fluids may be helpful to identify patients at high risk of metachronous peritoneal metastases."
Comment 10: Lines 159-160: Please provide an explanation for CCR abbreviation.

Response: Thank you for your careful review and correction. The correct abbreviation is “CC”, which has been defined.

VERSION 2 – REVIEW

Reviewer(s)' Comments to Author:

We are awaiting the receipt of the Digital Reader for the Nomogram.

We also need a "language review" and have other items for the authors to address. Not ready for final decision now.

VERSION 2 – AUTHOR RESPONSE

Dear editor,

We have put the digital nomogram in the legend of Fig3(Line 437). The website of it is https://qxssysu.shinyapps.io/CRCSPM_OS_DynNomapp/. If you have better suggestions, we are very willing to make further modifications.
